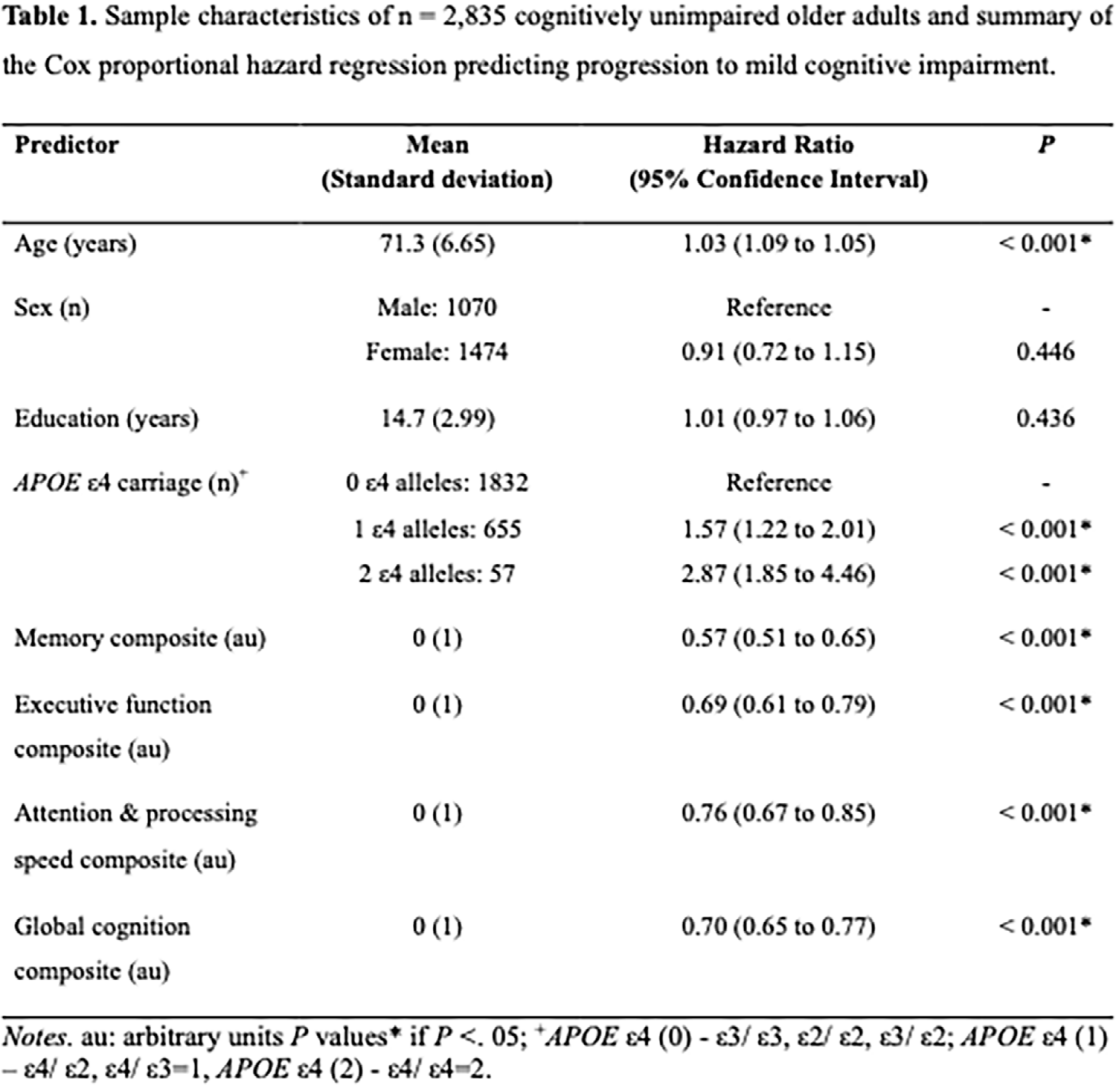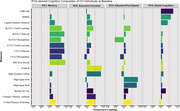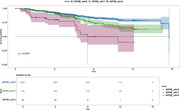# Data‐driven Cognitive Composites and *APOE* ε4 Carriage Predict Progression to Mild Cognitive Impairment in Cognitively Unimpaired Older Adults

**DOI:** 10.1002/alz.090147

**Published:** 2025-01-03

**Authors:** Bhargav Tallapragada, Brook Galna, Rosita Shishegar, Shaun J. Markovic, Belinda M. Brown, Vincent Dore, Simon M. Laws, Eleanor K. O’Brien, Tenielle Porter, Samantha C. Burnham, Michael S. W. Weiner, John C. Morris, Jason J. Hassenstab, Christopher C. Rowe, Liang Jin, Jurgen Fripp, Yen Ying Lim, James D. Doecke, Colin L. Masters, Paul Maruff, Hamid R. Sohrabi, the ADOPIC Consortium

**Affiliations:** ^1^ Centre for Healthy Ageing, Murdoch University, Murdoch, Perth, Western Australia Australia; ^2^ Centre for Molecular Medicine and Innovative Therapeutics, Perth, Western Australia Australia; ^3^ Turner Institute for Brain and Mental Health, School of Psychological Sciences, Monash University, Melbourne, VIC Australia; ^4^ The Australian e‐Health Research Centre, CSIRO, Parkville, VIC Australia; ^5^ School of Arts and Humanities, Edith Cowan University, Joondalup, Western Australia Australia; ^6^ Edith Cowan University, Perth, Western Australia Australia; ^7^ Murdoch University, Perth, Western Australia Australia; ^8^ Alzheimer’s Research Australia, Perth, Western Australia Australia; ^9^ The Australian e‐Health Research Centre, Commonwealth Scientific and Industrial Research Organisation, Melbourne, VIC Australia; ^10^ Curtin Medical School, Curtin University, Bentley, Western Australia Australia; ^11^ Centre for Precision Health, Edith Cowan University, Joondalup, Western Australia Australia; ^12^ Eli Lilly and Company, Indianapolis, IN USA; ^13^ Avid Radiopharmaceuticals, Philadelphia, PA USA; ^14^ University of California, San Francisco, San Francisco, CA USA; ^15^ Washington University in St. Louis, School of Medicine, St. Louis, MO USA; ^16^ Knight Alzheimer Disease Research Center, St. Louis, MO USA; ^17^ Washington University School of Medicine, St. Louis, MO USA; ^18^ Departments of Medicine and Molecular Imaging, University of Melbourne, Austin Health, Melbourne, VIC Australia; ^19^ Florey Institute of Neuroscience and Mental Health, University of Melbourne, Parkville, VIC Australia; ^20^ CSIRO Health and Biosecurity, Australian E‐Health Research Centre, Brisbane, QLD Australia; ^21^ The Australian e‐Health Research Centre, CSIRO, Brisbane, QLD Australia; ^22^ Cogstate Ltd., Melbourne, VIC Australia

## Abstract

**Background:**

In cognitively unimpaired (CU) individuals, the PACC is widely used as a cognitive outcome measure and endpoint in observational studies and clinical trials. However, it has drawn criticism for being heavily weighted towards memory. Increasing evidence indicates a decline spanning multiple cognitive domains in CU individuals. Therefore, using principal component analysis (PCA), we derived data‐driven domain‐specific cognitive composites. And subsequently, compared them against their summed z‐score counterparts in predicting progression to mild cognitive impairment (MCI).

**Method:**

Baseline cognitive, demographic, and genotype data of 2,853 CU older adults (aged 41.6 to 98.3) was obtained from the Alzheimer’s Dementia Onset and Progression in International Cohorts (ADOPIC) Consortium. Using varimax‐rotated PCA, tests significantly loading (≥ 0.5) onto each principal component were extracted to derive domain‐specific cognitive composites. The resulting domain scores were normalised to a mean of 0 and SD of 1, with a higher score indicating better cognition. Cox regression was used to assess the association between progression to MCI and baseline demographics, cognition, and *APOE* ε4 carriage. Akaike information criteria (AIC) was used to compare the fitness of PCA‐derived composites against the zPACC and z‐score domain‐specific composites.

**Result:**

Baseline cohort characteristics are described in Table 1. PCA explained 68% of the variance and resulted in four independent cognitive composites (Figure 1): memory; executive function; attention and processing speed; and global cognition. At 15 years from baseline, 309 participants progressed to MCI, while 2,544 remained CU. Cox regression showed that the four cognitive composites, age and *APOE* genotype significantly predicted progression to MCI (Concordance = 77%, *p* < 0.001, AIC = 4105, Table 1). Additionally, the PCA‐derived composites performed comparably, if not better than the summed z‐score counterparts, the PACC (Concordance = 74%, *p* < 0.001, AIC = 4163) and domain‐specific composites (Concordance = 75%, *p* < 0.001, AIC = 4142). Baseline older age, *APOE* ε4 carriage in a dose‐dependent manner (Figure 2) and poorer cognition for each PCA‐composite were independently associated with progression to MCI.

**Conclusion:**

Together with *APOE* ε4 carriage, our PCA‐derived domain‐specific composites performed better than their summed z‐score counterparts at predicting progression to MCI 15 years before symptom onset.